# Text-to-image generation with enhanced GANs: Bridging semantic gaps using RNN and CNN

**DOI:** 10.1371/journal.pone.0340413

**Published:** 2026-01-21

**Authors:** Sadia Ramzan, Hafiz Arslan Ramzan, Tehmina Kalsum, Mohammed Adnan, Muhammad Akmal Chaudhary, Muhammad Moazzam Ali

**Affiliations:** 1 Department of Computer Science, Emerson University, Multan, Pakistan; 2 School of Electrical Engineering and Computer Sciences, National University of Sciences and Technology, Islamabad, Pakistan; 3 Departments of Software Engineering, University of Engineering and Technology, Taxila, Pakistan; 4 Departments of Electrical and Computer Engineering, Ajman University, Ajman, United Arab Emirates; Nanchang University, CHINA

## Abstract

Text-to-image generation is the process of generating images from a given text description. It is the most challenging task to produce consistently realistic images according to our conditions. We have considered this problem in our study and proposed a neural network-based model that can generate good-quality images from text descriptions. In this research, we have used a Generative Adversarial Network (GAN) for the generation of images with Recurrent Neural Network (RNN) and Convolutional Neural Network (CNN). RNN is used for creating word embeddings from textual sentences and for extracting important features from images we have used CNN. The generator model is used for generating images from text and this generated image is used as input to the discriminator with further matched text, mismatched text, and real images from the dataset. These experiments are performed on the Oxford 102-flowers dataset. We also modified this existing dataset and created a new version of this dataset, oxford-102 flowers (beta) consisting of 15 text descriptions for each image. The model is trained on these two datasets for generating images of 64 x 64, 128 x 128, and 256 x 256 resolution. Generator and discriminator loss during training of mode are calculated. The inception Score and peak signal-to-noise ratio are performance metrics that we have used for model evaluation. Our model achieves an inception score of 4.15 on the oxford-102 flowers dataset of 64 x 64 resolution, 3.87 on 256 x 256 resolution, and 3.97 on 128 x 128 oxford-102 flowers (beta). PSNR values are 28.25 dB and 30.12dB on the original and annotated dataset. Experiments show the outstanding performance of our methodology as compared to the existing models in terms of inception score and PSNR value.

## Introduction

One of the most demanding and significant challenges with machine learning is creating pictures from textual information. This work entails employing techniques of natural language processing (NLP) and algorithms to solve language diversity issues, such as controlling and managing partial and ambiguous information. Following that, computer vision methods and algorithms use this data to learn. It is currently one of the most recent computer vision research topics.

When people read or listen to a narrative, They eventually generate images in their minds to visualize the contents. We seldom think about our capacity to envision and comprehend the complex link between language and the chromatic world since it is so intuitive. Several cognitive processes, including memory, reasoning, and thought processes, depend on mental visual imagery or “seeing with the mind’s eye.” [1]. A significant step toward improving user intellectual capacity is the development of technology that can transform written descriptions into images and recognize the relationship between vision and words.

**Fig 1:**
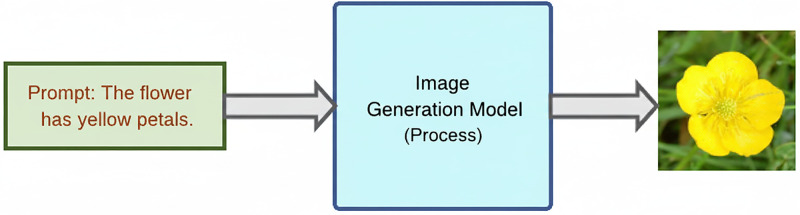
Text to image generation.

Recent developments facilitated by the evolution of deep learning have led to a huge advancement in computer vision applications and image processing techniques. The generation of images from text is the domain of one of these expanding fields. The term “text-to-image” (T2I) refers to the process of creating a visually realistic photo that relates to a given written description. Referred to as image-to-text generation, or I2T [2–4], T2I generation is the opposite process of captioning an image that generates a text description from an image. The T2I generation model generates an RGB image that corresponds to the written description given as input. With the given text input “this flower has yellow petals,” the model generates a picture of a flower with yellow petals, as shown in Fig 1.

The study of T2I generation has been crucial in both computer vision and natural language processing due to its tremendous capability in a variety of applications. Photo-searching, photo-editing, computer-aided design, industrial design, art creation, image reconstruction, live captioning, portrait drawing, cross-modal retrieval, and image manipulation are a few common applications for producing photo-realistic visuals from text. Because of its various practical uses, image generation is a fascinating and significant effort. Generative Adversarial Networks (GAN) were first introduced in a study released in 2014 by Ian Goodfellow and a group of University of Montreal scholars [1], especially Yoshua Bengio. Yann LeCun, Facebook’s AI research director, declared adversarial training “the most fascinating notion in machine learning in recent years”. The development of GANs has shown remarkable effectiveness in data augmentation, image-to-image conversion, style transfer, image synthesis, and image super-resolution.

Generative Adversarial Networks (GANs) are deep learning models based on convolutional neural networks (CNNs) [5,6]. They consist of two neural networks, a generator that creates data and a discriminator that distinguishes real from fake samples, trained through a game-theoretic framework to improve generation quality [7,8]. Unlike discriminative models that classify input data, generative models aim to produce new, realistic samples. A key challenge lies in capturing the semantics, context, and relationships within textual input to generate accurate and interpretable images. Controlling this process to match desired features or styles requires conditioning generation on both textual input and control variables. Using the Oxford-102 Flowers dataset, we addressed these issues by expanding data diversity, reducing text-image matching bias, and refining training to mitigate mode collapse. Improved model design enhances semantic alignment, image diversity, and computational efficiency.

- One of the main contributions is the preparation of a newer version of the existing oxford-102 flowers dataset. Originally this dataset consisted of 8189 images of flowers and 102 classes. To achieve better results, we modified its annotations and named it the Oxford-102 Flowers (beta) version. This version of the dataset contains 15 text descriptions for every single image.
- Generation of images with 64 x 64 size of resolution and 256 x 256 resolution.
- We used a standard performance metric PSNR in GAN for testing the quality of the generated image. The results show the improved PSNR and inception score values of images generated by text.

The rest of the paper is organized as follows: The research that has already been done utilizing various deep learning algorithms is covered in detail in Literature Review Section. This part offers a critical analysis of earlier text-to-image-generating efforts. In Proposed Methodology Section, we go through the specifics and the procedures used to assess the proposed works. Each circumstance that helped us develop the framework in the article is described in great detail below. The suggested work is given an extensive analysis. In Performance Evaluation Section, we go over the experimentation limitations, the outcomes of the approach as proposed, and the next work. Conclusion Section of the paper summarizes our findings and addresses our recommendations for further research and future work Table 1.

**Table 1:** Abbreviations.

Keywords	Abbreviations
GAN	Generative Adversarial Network
T2I	Text to Image
DL	Deep Learning
ML	Machine Learning
CLS	Conditional Latent Space
INT	Interpolation
DNN	Deep Neural Network
CNN	Convolutional Neural Network
RNN	Recurrent Neural Network
ResNet	Residual Network
LSTM	Long-Short Term Memory
DCM	Detailed Correction Module
ACM	Affine Combination Module
IS	Inception Score
PSNR	Peak Signal to Noise Ratio
VS	Visual semantic Similarity
FSD	face semantic distance
MSE	Mean Square Error
VrR-VG	Visually-relevant relationships

## Literature survey

Although Goodfellow [1] initially introduced GANs in 2014, Reed et al. [2] were the very first to use them in the creation of images from text in 2016. Their studies produced valuable images dependent on the description in the text. Text-to-image generation is performed on multiple datasets, their literature survey is classified by used dataset.

Reed et al. [2] developed a GAN-based architecture for characters to pixels translation of visual concepts. The purpose of their study is to convert the text description that is a human-written single sentence directly into an image pixel. The distinction of their model is that instead of working on class labels, they conditioned their model on the text description. They used Oxford-102 flowers [2] and Caltech-UCSD Birds datasets [4] with human written five text descriptions per image. Additionally, the model has also been applied to the MS COCO dataset [5]. According to experimental results, their model can synthesize text captions into plausible visual interpretations. Khan et al. [6] proposed a fully trained GAN for the generation of natural and realistic text-to-face images. The image decoder and text encoder are both taught at the same time to provide more precise and effective results. They proposed two discriminators for exploiting the power of joint learning. In addition to fully trained GAN for text-to-face image synthesis, they also contributed to dataset generation. Moreover, their methodology also presents the similarity details between input sentences ground-truth and the generated face images. Their proposed model generates good-quality images according to given input sentences.

Mishra et al. [7] aim to produce reasonable-quality images of flowers by developing a Residual GAN or RGAN. The motivation of this work is based on RNN and CNN which extract the important information from text. Oxford-102 flower dataset [2] is used with CNN which will give vector embedding’s of images. Text-to-image architecture is presented that utilizes skip connection with learning rate decay processes that reach the faster convergence, stabilize the training process, and result in the generation of detailed text descriptions. Results show that conditioned GAN can produce several images according to text description which is the natural multi-modality in GAN. Ouyang et al. [8] generated real-life images by proposing a neural network architecture of LSTM Conditional GAN. Caltech-UCSD-200–211 [4] and Oxford-102 flowers [2] datasets are trained on the proposed model. The proposed methodology outperforms the state-of-the-art approaches and produces better results. Li et al. [9] proposed a model for text-to-image generation consisting of two components: a detailed correction module (DCM) and an affine combination module (ACM). They aim to edit image parts semantically according to the text input given by the user, whereas the other parts of the image that are not described in the text remain the same. Only low-quality images can be produced by text-guided image manipulation. They introduced a novel approach of GAN for creating high-quality images by text-guided image manipulation. CUB [4] and COCO datasets [5] are used for the evaluation of the model. Results show that the trained model generates high-quality and effective image manipulations.

Knowledge Transfer GAN (KT-GAN) [10] is the integration of the mechanisms of semantic distillation (SDM) and attention transfer (AATM). These two techniques identify the key details of an image from words and enable the generator to produce a high-quality picture. Experiments are performed by measuring FID and Rank-1 on MS COCO and CUB datasets, which show that KT-GAN outperforms the previous methods. Zhang et al. [11] worked on the separation of foreground and background for high-quality text to image generation. They proposed a context-aware conditional Variation Auto encoder (CVAE) for capturing the text-based color and basic layout of images, which makes more effective text-image alignment by differentiating between background and foreground of images. For realistic image synthesis, the generation of VAE is refined by conditional GAN, which corrects the defects and recovers the lost details. Results from the experiments show that the suggested model has less variation and is more stable, and it outperforms the two compared methods (StackGAN and GAN-INT-CLS) on CUB and Oxford-102 flowers [2].

Zhang et al. [12] introduced network hierarchical-nested adversarial objectives and multipurpose adversarial loss. They worked on two major difficulties, convergence balancing and guaranteeing semantic consistency. They evaluated their proposed method on three datasets, MSCOCO, CUB birds, and Oxford-102-flowers. Their results show that their proposed methodology can capture better graphic semantic statistics in produced images. Object-driven GAN (Obj-GAN) [13] synthesizes images from the text for complex scenes. At first, the proposed Obj-GAN encodes the whole text into a single sentence vector, and a fast discriminator is proposed based on R-CNN [14,15] which provides rich discrimination signals. According to Sharma et al. [16], text descriptions are not enough for synthesizing good-quality images, that’s why they added dialogue for further describing the scenes so that the quality of generated images gives a better IS. They used the MS COCO dataset, and their model gives a higher IS as compared to StackGAN.

Similarity-aware discriminators and multi-stage attention-modulated generators also known as MS-GAN [17] use multiple convolutional blocks in the generator which are modulated by locally and globally attended features that are calculated between text and output image. During text to image conversion, semantic information is better preserved by a generator with attention-modulation. In order to preserve semantic coherence within the produced image and text description a similarity aware discriminator is proposed. Experimental results produced better performance on MS-COCO and CUB datasets.

For text-to-image synthesis [18], the authors suggest a novel architecture called T2IGAN, which is automatically explored via neural architecture search (NAS). Furthermore, a lightweight transformer is used in our search space to effectively merge the text features and picture characteristics, taking into account the remarkable powers of the well-known transformer in computer vision and natural language processing. Finally, experimental evaluation on the common text-to-image synthesis datasets demonstrates the extraordinary efficacy of our searched T2IGAN. The Multi-sentence Auxiliary Generative Adversarial Networks (MA-GAN) approach [19], which the authors propose for text-to-image synthesis, guarantees the generation similarity of related sentences and improves generation quality by investigating the semantic correlation between different sentences describing the same image. To improve the dependability of the produced findings and lessen variance between their generated pictures, they explicitly suggest a Single-sentence Generation and Multi-sentence Discrimination (SGMD) module that investigates the semantic connection between many related phrases. Additionally, a process called Progressive Negative Sample Selection (PNSS) is intended to extract more appropriate negative samples for training. This may help the generative model achieve detailed discrimination and provide more refined output.

In order to address the issue of global coherent patterns in complicated sceneries, the authors suggest a model known as CapGAN [20], which creates graphics from a single text sentence. The provided text is encoded into a vector form for this purpose using skip-thought vectors. The encoded vector is used as an input for the adversarial process of picture synthesis, whereby the generator (G) and discriminator (D) models are concurrently trained. While model D attempts to forecast the sample using training data instead of using pictures created by model G, model G creates fictitious images. The use of integrating capsules at the discriminator level to help the model comprehend the relative spatial relationships and orientations between various elements of an object in an image is the conceptual innovation of this study [21].

Current studies on text-to-image generation using GANs encounter methodological restrictions. The small size of the dataset, obstacles to diversity and generalization, difficulties matching verbal descriptions with photos, and the difficulty of bridging the semantic divide between text and image content are a few of these. Semantic relevance may not be fully captured by evaluation criteria such as Inception Score. Exploration of complicated designs is hampered by GANs’ high computational resource requirements and tendency toward mode collapse, which reduces diversity. Moreover, the small details necessary in generating fine-grained images, such particular flower species, continue to be difficult.

Using the Oxford-102 Flowers dataset, we have tackled a number of significant text-to-image generation challenges. First off, by supplementing the dataset with more pictures and textual descriptions, we have greatly expanded its size, which has improved variety and our models’ capacity for generalization. Second, we have improved the techniques for correctly matching textual descriptions with photos, which has decreased biases and inconsistencies in the dataset. Furthermore, our unique model design integrates state-of-the-art methods for bridging the semantic gap between text and image information, leading to more realistic and accurate image production. We have successfully addressed mode collapse difficulties and enhanced the overall diversity and quality of the generated images by utilizing cutting-edge evaluation measures and optimizing our training procedures. Furthermore, our study has enhanced computational.

In summary, existing models demonstrate significant progress in text-to-image synthesis but still face limitations such as semantic misalignment between modalities, insufficient diversity, and unstable training at higher resolutions. The proposed Recurrent Convolutional GAN (RC-GAN) model directly addresses these challenges by integrating RNN-based contextual encoding with CNN-based visual refinement, supported by an enriched dataset to enhance textual diversity and generalization.

## Proposed methodology

In this study, we have used GANs with RNN and CNN, for producing meaningful visuals from a written description. Along with these deep learning algorithms we have used TensorFlow, Tensorlayer, NLTK, and NumPy. The used dataset consists of different images of flowers with their respective text descriptions. To convert input texts into realistic images using GAN, firstly we perform preprocessing of text and image data. We take text descriptions from the dataset, and preprocess these caption sentences by creating vocabulary. After that, the list stores these captions along with their corresponding IDs. The downloaded dataset contains images with multiple dimensions, so for better training images are loaded and resized to a fixed dimension of 128. This pre-processed data is divided into training and testing sets. For generating images with 64 x 64 and 128 x 128 resolution we used 8000 training images with 80000 text descriptions used as input to the proposed model.

Text-to-image mapping is performed by using RNN and CNN. Training set captions are used as input to RNN encoder for converting them to text embedding. The RNN takes input sequence and converts the textual descriptions into word embedding of size 256, text feature dimensions 128. CNN is used for extracting features of real images and mismatched images.

**Fig 2:**
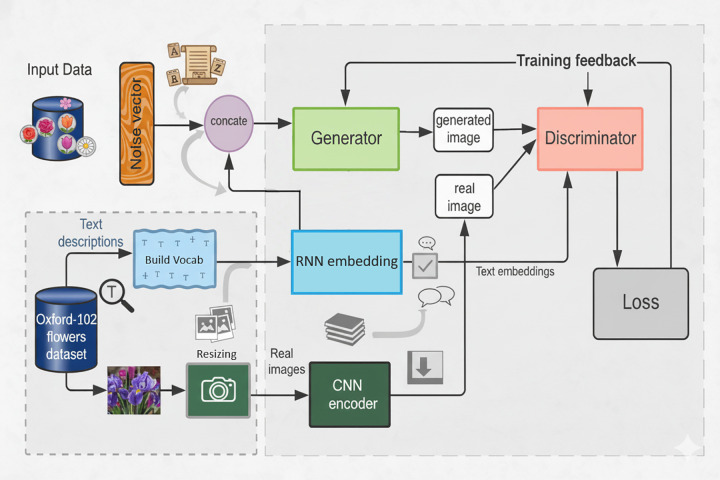
Overall architecture of the proposed RC-GAN model showing integration of RNN for text embedding and CNN for visual feature extraction.

For generating images from text mainly we use a couple of neural networks that are generators and discriminators as shown in Fig 2. The generator produces images from text input and the output of the generator is further used as input to the discriminator.

In order to address the requirement for a major improvement in our proposed hybrid model named Recurrent Convolutional GAN (RC-GAN), we have rigorously explored a number of approaches. To fully utilize the advantages of each component, the model has undergone painstaking development. We have applied advanced training techniques to improve the model’s convergence and learning ability. To ensure strong training and generalization, the dataset has been augmented to increase its scope and diversity.

Unlike prior GAN variants such as StackGAN, MA-GAN, or DF-GAN, which primarily focus on hierarchical or attention-based refinement, the proposed RC-GAN introduces a recurrent-convolutional integration that strengthens cross-modal semantic alignment between text embeddings and image features. This hybrid coupling enables the model to retain contextual consistency across resolutions and reduce mode collapse. In contrast to StackGAN and HDGAN, RC-GAN jointly optimizes both textual embedding and visual feature extraction, improving generative stability and diversity.

We collected a large dataset of matched textual descriptions and photos, covering a variety of pertinent categories, to improve image quality and diversity. While photos were resized and normalized, textual descriptions were preprocessed using tokenization. To boost dataset diversity, the data set is expanded by including more textual descriptions for each image. To establish training examples, each description was precisely matched with the matching image, guaranteeing alignment and consistency. By these efforts, we were able to improve the dataset so that the hybrid model could efficiently produce images of high quality.

In addition, our assessment techniques have been thoroughly improved to offer thorough evaluations of the model’s functionality. By means of these coordinated endeavors, we have confirmed the legitimacy and effectiveness of our suggested hybrid approach in producing varied and superior visuals from written descriptions.

### Generator

The word embedding from RNN is paired with a 512-dimensional noise vector [selected from a latent space that is generally a random Normal distribution] and given as input to the generator model. We use gated-feedback 128 with a batch size of 64.

Combined text image input is further given to the dense layer, batch norm layer and reshape layer. Output from the previous layer is used for convolution. CNN’s key benefit is that it recognizes useful characteristics without the need for human intervention.

### Discriminator

A series of unique (image, text) pairs are given to the model as input during training to achieve the Discriminator’s objective:

- Input pair of (Real Image, Real Text Description)
- Input pair of (Wrong Image, Text Description)
- Input pair of (Fake Image, Text Description)

To make it possible for the model to figure out whether a specific picture and text combination aligns properly or not, the actual combinations of text and actual photo are required. The inaccurate image, as indicated by the Real Text Description, shows that the picture and the caption are not the same. The goal variable is set to 0 in this example, so that the model may learn that the picture and description are not aligned. In this example, False Image refers to an image created by the Generator; the target variable is set to 0 for the Discriminator model to discriminate between genuine and fake images.

RNN loss = rnn_embed(real caption), rnn_embed(wrong caption)
CNN loss = cnn_encoder(real image), cnn_encoder(wrong image)
D loss = real image + (generated image + mismatched image)/2
G loss = (fake-image_logits)

Training is performed on oxford-102 flowers dataset and our created oxford-102 flowers (beta) which includes images with 15 alternative written descriptions that explain the characteristics of each image. We have used a learning rate of 0.002 with alpha 0.5 and a learning rate decay 100. RNN loss is adjusted to 0 after the first 50 iterations. We take sample seed sentence equal to batch size which is 64 in our training. For 64 x 64 image resolution we generate a grid of images containing 8 x 8 images. We also generated images of 128 and 256 resolution.

### Dataset

**Fig 3:**
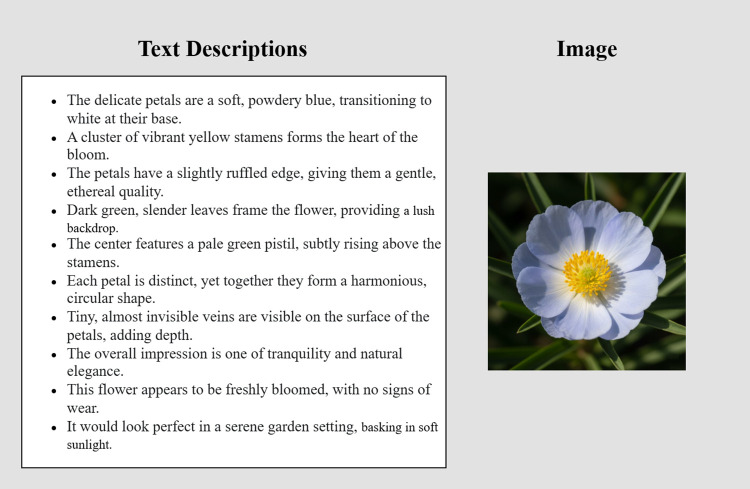
Original dataset image with 10 descriptions.

The Oxford-102 flowers dataset, which consists of 8189 images of different kinds of flowers, is used. There are 102 classes in all, with 40–60 photos in each class and 10 written descriptions for each image. In this study, 8000 images were taken into consideration for training. Using this dataset, the model is trained for 200 epochs. An example from oxfrd-102 flowers is taken consisting of an image and respective ten text description shown in Fig 3.

**Fig 4:**
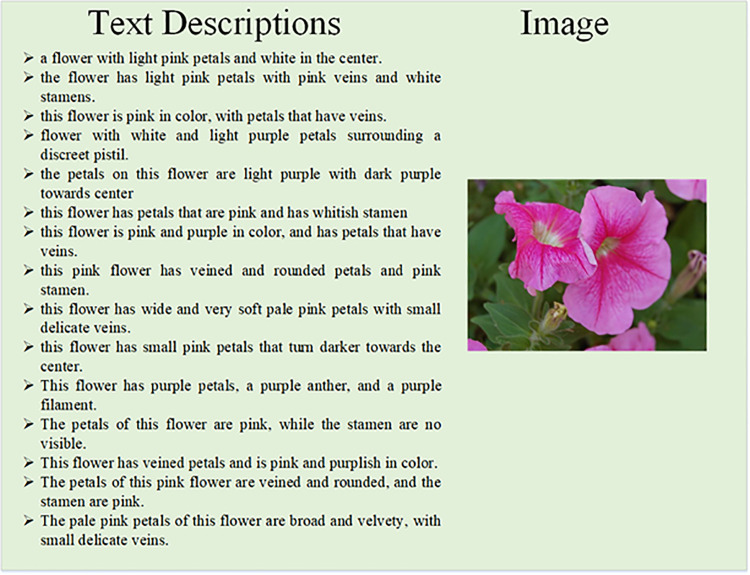
Beta dataset image with 15 descriptions.

Oxford-102 flowers (Beta) is the updated version of oxford-102 flowers dataset, which contains of 8198 images of flowers and each image consisting fifteen text descriptions as shown in Fig 4.

For the Oxford-102 Flowers (Beta) dataset, additional textual annotations were generated through a hybrid process combining manual curation and NLP-based text augmentation. Initially, five human annotators provided ten descriptions per image, focusing on color, petal shape, and texture attributes. Subsequently, five supplementary captions were generated using a pretrained GPT-2 language model fine-tuned on floral descriptions, which were manually verified for semantic accuracy and grammatical quality. This approach ensured reproducibility while enhancing descriptive diversity.

### Data pre-processing

**Fig 5:**
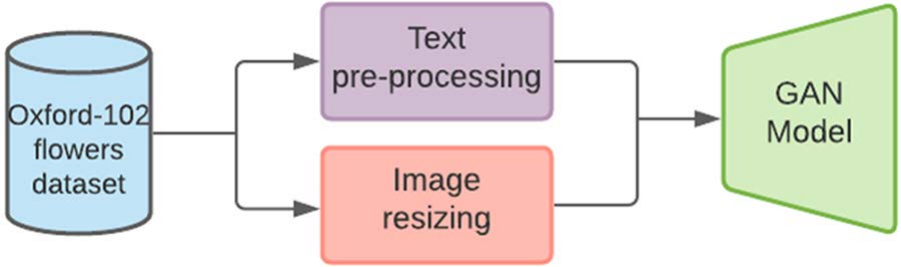
Data preprocessing and data loading.

With the initial collection and extraction of the data, it contains 8189 images with various resolutions and associated text descriptions. NLTK tokenizer has been used to normalize textual data, resulting in the conversion of textual phrases into words. The array of caption IDs is created from lists of these tokenized words. Images are loaded and resized to the same proportions. All the available training and testing images has been resized to 128 x 128 pixels in resolution. These captions’ vocabulary and all images are saved in pickle format. For training purposes, the pickle files of images are transformed into arrays and both images and vocab are loaded to model. Fig 5 shows the pre-processing and resizing steps of the data.

**Algorithm: Data loading and pre-processing**

Input: Oxford-102 Flowers (text, images)

Output: Pre-processed text descriptions and resized images

Start

dataset = Oxford-102 flowers

if (dataset==102 flowers):

Captions⇓list_captions

Vocab⇓ preprocessed captions

Img_capt = captions_dict

Tokenize(Vocab)

Images = (load image_dir)

for images = list do:

img_r = imresize(images)

end for

end if.

### Training algorithm

The training algorithm of the proposed model is described here, which consists of matched text mt, mismatched text, real image from the dataset, the generated image from the generator model and batch size of 64. Its training starts with a loop of batch size 64 and mini-batch size of 125. Real text description is used as input to the generator for the generation of the corresponding image. The generated image mi^ is further used as input to the discriminator with matched text mt and mismatched text mt^ and real image from dataset. The discriminator output of generated image mt^ with matching text mt is used for calculating the generator loss (G_loss). The discriminators output of generated image and real description, real image and mismatched descriptions are added, and combined with real image and real descriptions, and their output is divided by 2 for calculating the discriminators loss (D_loss). According to G_loss and D_loss the generator and discriminator weights are updated for every iteration.

**Algorithm**: **RC-GAN training algorithm with batch size 64**

**Input** (matching text mt, real image mi, mismatching text mt^, number of training batch steps N)

**Output**: The trained Generator G and discriminator D

Start

N = 64

**for** n = 1 to N **do:**

**for** m = 1 to minibatch **do**:

encode mt

encode mt^

Draw ‘S’ sample of random noise

mi^ = G ⇓ forward (S, mt)

D_FR_⇓ mi^, mt

D_RR_⇓ mi; mt

D_RW_⇓ mi; mt^

G loss = (D_FR_.output)

D loss = D_RR_.output + (D_FR_.output + D_RW_.output)/ 2

Fix the generator G

Update discriminator D

Fix the discriminator D

Update generator G

end for

end for

#### RNN.

Recurrent Neural Network, or RNN for short, is a kind of neural network design that combines the best features of recurrent and convolutional neural networks (RNN and CNN, respectively). Sequence data, including audio, text, and picture data, is often processed and classified using Convolutional Recurrent Neural Networks, or RNNs for short. They perform especially well in tasks requiring the comprehension and modelling of temporal and contextual information because of their capacity to handle variable-length sequential input and grasp long-term dependencies. RNNs exhibit optimal performance on a range of tasks and are very effective instruments for sequential data processing and modelling. RNNs function as follows: An RNN receives a series of data as input, which might be audio samples or pictures. Convolutional layers are used to extract features from the input sequence. Especially useful for image-based inputs, these layers resemble those found in CNNs. Afterwards, one or more recurrent layers, which work especially well when processing sequential data,feed the output of a convolutional layer. Recurring layers preserve a concealed state that contains details about earlier sequence entries.

Prior to being fed into a recurrent layer, the output of a convolutional layer is typically sampled. This preserves important input features while lowering the computational complexity of the network. After passing through the fully linked final layer, the output of the last iterative layer generates a forecast for the input sequence. This forecast might be a string of letters, a word, or another task-related output.

#### CNN.

One kind of deep learning neural network technique that is often employed in the picture classification process is the CNN, or convolutional neural network. Deep learning algorithms have shown impressive results in a range of computer vision tasks in recent times. To carry out the convolution process by generating a kernel based on the data section of the input data, CNN incorporates a convolutional layer in its application. The pooling layer subsequently processes the convolutional layer’s output. By using data that has been decided by the formula on certain data pieces, such as the average value or the maximum value, it is possible to minimize the quantity of data without losing key characteristics. The sum of the convolutional and pooling layers is then used to repeat this procedure. To create a single lengthy dimension, the data is then typically flattened. In the last stage, the data is sent into the fully connected layer for classification, where it receives the output of the CNN process along with the right label.

### Libraries

TensorFlow a ML library which is developed by Google is used with Python and JavaScript for making computations and training models. It is an open-source library that makes computations faster and develops algorithms utilizing data flow graphs. It is most frequently used for classification, discovery, perception, prediction, comprehension, and production. TensorLayer is a deep learning library based on TensorFlow. It is a high level, very simple, and easy-to-understand model which is used for building training complex artificial intelligence models very quickly.

To create new, synthetic samples of data that can be passed for real-world information GANs are used, GANs are computational structures that pit two neural networks against one another (thus the name “adversarial” is employed. Batch normalization technique used in deep learning models to combat the internal covariate shift problem, optimizing the performance and speedy learning. It stabilizes the learning process, normalizes network activations throughout a predetermined mini-batch, and greatly lowers the quantity of the training epochs needed to construct deep networks. Layer normalization is used for normalizing the distributions of intermediate layers. Convolutional layers learns the characteristics of an image from small input data squares, preserving the relationship between pixels. The mathematical technique requires two inputs: an image matrix and a filter or kernel. By addressing degradation difficulties through skip connections, residual networks allow very deep networks to be built without experiencing reduction in performance. Neural networks’ dense layer makes it easier to perform highly linked calculations; CNN models primarily employ it for matrix-vector multiplication. Like RMSProp and AdaGrad, the Adam optimizer is simple to set up and has default settings that work well in most situations. It offers an adaptive solution for noisy circumstances with sparse gradients.

## Performance evaluation

For assessing any model some metrics are used that are known as performance metrics. Performance metrics that are mostly used for the evaluation of GANs are, i.e., Frechet Inception Distance, Average log-likelihood, Inception Score, PSNR, etc. For evaluating our model, we have used IS and PSNR.

### Inception score

Now a day’s evaluation of generative models is a very interesting research domain. A recently proposed technique that is employed to evaluate the quality level of generated images, specifically the synthetic images generated by GAN models is IS. Its name is derived from the Google inception classifier and was proposed by Tim Salimans et al. [22]. It is employed to categorize a massive number of generated images. The objective of the score is to capture two characteristics of a set of created images: quality of images, and diversity of images.

The conditional probability grabs our attention in image quality, and all created photos in the collection need to have a low entropy conditional probability. The entropy is derived by multiplying the probability’s log to the observed probability of each negative sum. It is believed that larger probabilities contain less information than smaller probabilities.


Entropy = −sum (p_i * log (p_i)) (1)

For calculating the IS for a group of photos, it is necessary to determine each image’s conditional probability (p(b|a)) using the inception v3 model. The marginal probability is then calculated using the average of the conditional probabilities for every picture in the group (p(b)). The KL divergence is the product of the conditional probability and the log of the conditional probability minus the log of the marginal probability for every picture.


KL divergence = (log (p(b|a) – log (p(b)) * p(b|a) (2)

The KL divergence is then aggregated across all photos and averaged across all classes, with the result’s exponent, which is calculated to produce the final score.

### PSNR

The PSNR is a performance metric that is utilized to determine decibels representing of highest signal-to-noise ratio, between two photos. This ratio is utilized to evaluate the quality of the generated and original images. When the quality of the generated or synthesized image improves, the PSNR value rises. The values of PSNR are calculated using the following equation.


$$PSNR\;=\;\text{10}\;\text{log10}\;({\text{R}}^{\text{2}}\;/\;\text{MSE})$$ (3)

In addition to IS and PSNR, we computed the Fréchet Inception Distance (FID) and Human Rank (HR) scores to capture perceptual and semantic alignment. FID was calculated using Inception-V3 activations from 10,000 generated and real images. A lower FID indicates a closer distributional similarity between generated and ground truth images. Furthermore, a human ranking study involving 20 participants evaluated realism and semantic relevance. Statistical significance between baseline models and RC-GAN results was verified using independent t-tests (p < 0.05), confirming the robustness of improvements.

### Comparitive analysis

**Fig 6:**
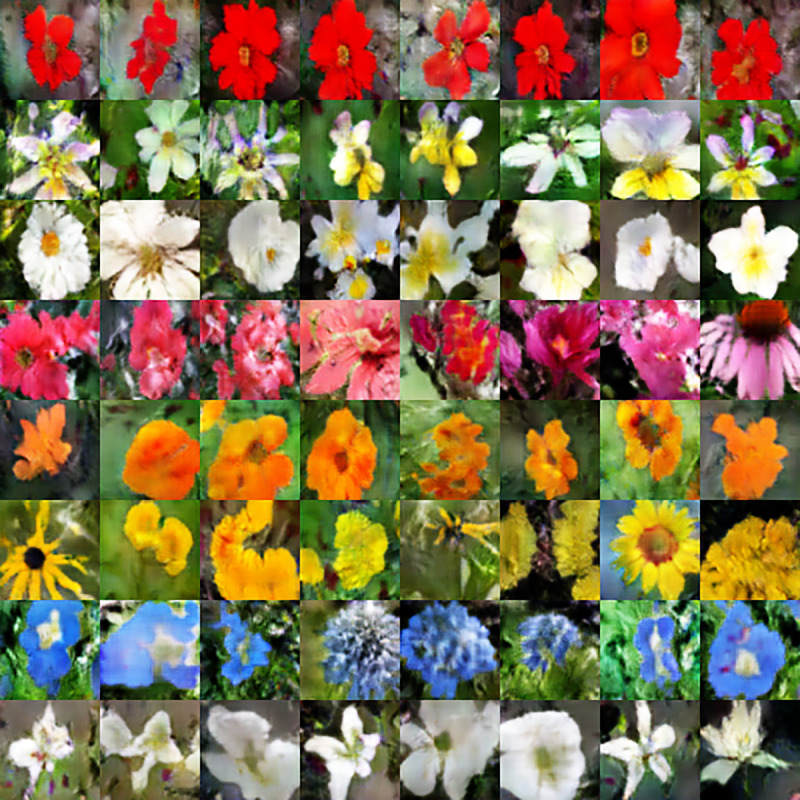
Generated image of 64 x 64 dimensions.

This subsection encompasses the details regarding the implementation of T2I generation with the details of experimentation performed on two data sets, i.e., Oxford-102 flowers and Oxford-102 flowers (Beta). These two variations of the dataset are used to completely assess the proposed model by fine-tuning the state-of-the-art deep learning frameworks. Results generated by our proposed method with different text descriptions and different resolutions are presented here. We have synthesized images of 64 x 64, 128 x 128, and 256 x 256 resolution sizes. Some of the output images that are generated by our trained model from input text are shown here. Fig 6 shows images of 64 x 64 resolution generated from multiple text descriptions.

Using the Oxford-102 flowers dataset, We compared our proposed model’s performance to the state of the art models, such as DualAttn-GAN, StackGAN, HDGAN, StackGAN++, T2IGAN, MA-GAN, SSBi-GAN and GAN-INT-CLS as show in Table 2. It presents an in-depth analysis of the Inception Scores attained by several models on the Oxford-102 Flowers dataset. Higher scores indicate better performance in producing realistic and aesthetically pleasing images. Inception Scores are a statistic used to assess the quality of created images.

**Table 2:** Inception Score of size 64 x 64, compared with state of the art models.

Ref.	Models	Dataset	Method	Inception Score
Reed et al. [1]	GAN-INT-CLS	Oxford-102 Flowers	Deep Learning	2.66 ± 0.03
Zhang et al. [23]	StackGAN	Oxford-102 Flowers	Deep Learning	3.20 ± 0.01
Zhang et al. [24]	StackGAN++	Oxford-102 Flowers	Deep Learning	3.26 ± 0.01
Zhang et al. [11]	HDGAN	Oxford-102 Flowers	Deep Learning	3.45 ± 0.05
Cai et al. [25]	DualAttn-GAN	Oxford-102 Flowers	Deep Learning	4.06 ± 0.05
Li et al. [18]	Lightweight Transformer(T2IGAN)	CUB-200 Birds and Oxford-102 Flowers.	Deep Learning	5.12 ± 0.01
Yang et al. [19]	MA-GAN	Oxford-102 and CUB	Deep Learning	4.09 ± 0.08
Tan et al. [21]	SSBi-GAN	Oxford-102 and CUB datasets	Deep Learning	N/A
Proposed Method	**RC-GAN**	Oxford-102 Flowers	Deep Learning	**4.15 ± 0.03**

The table highlights the wide range of deep learning approaches that different models apply to the goal of text-to-image generation. It demonstrates how well various methods work to produce high-quality images, as demonstrated by each method’s unique Inception Score. For a fair comparison, baseline scores were taken from the original papers where models were trained on the same Oxford-102 Flowers dataset at comparable resolutions. Where official results were unavailable, models were retrained under identical settings to ensure fairness.

Images are generated images with text descriptions from input text 1 to input text 8. These are images with a resolution of 128 x 128. The image below shows the image generated with a size of 256 in resolution.

Table 3 presents the inception score of 128 x 128 size resolution of images generated from proposed model. These images are generated by training model on the beta version of oxford-102 flowers dataset.

**Table 3:** IS on oxford-102 flowers (beta) of size 128 x 128.

Ref.	Model	Dataset	Method	Inception Score
Proposed Method	RC-GAN	**Oxford-102 Flowers (Beta)**	Deep Learning	**3.97 ± 0.02**

Inception score on oxford-102 flowers dataset with 256 x 256 size of images generated from our proposed methodology. With respect to Inception Score, the suggested RC-GAN model shows encouraging results. The Table 4 summarizes the performance of many deep learning models on the Oxford-102 Flowers dataset.

**Table 4:** Inception Score on oxford-102 flowers of size 256 x 256.

Ref.	Model	Dataset	Method	Inception Score
Zhang et al. [23]	StackGAN	Oxford-102 Flowers	Deep Learning	3.71 ± 0.04
Cheng et al. [26]	CWPGGAN	Oxford-102 Flowers	Deep Learning	3.76 ± 0.03
Li et al. [18]	Lightweight Transformer(T2IGAN)	Oxford-102 Flowers.	Deep Learning	4.89 ± 0.01
Reed et al. [2]	GAN-INT-CLS	Oxford-102 Flowers	Deep Learning	2.66 ± 0.03
Proposed Method	**RC-GAN**	Oxford-102 Flowers	Deep Learning	**3.87 ± 0.02**

**Fig 7:**
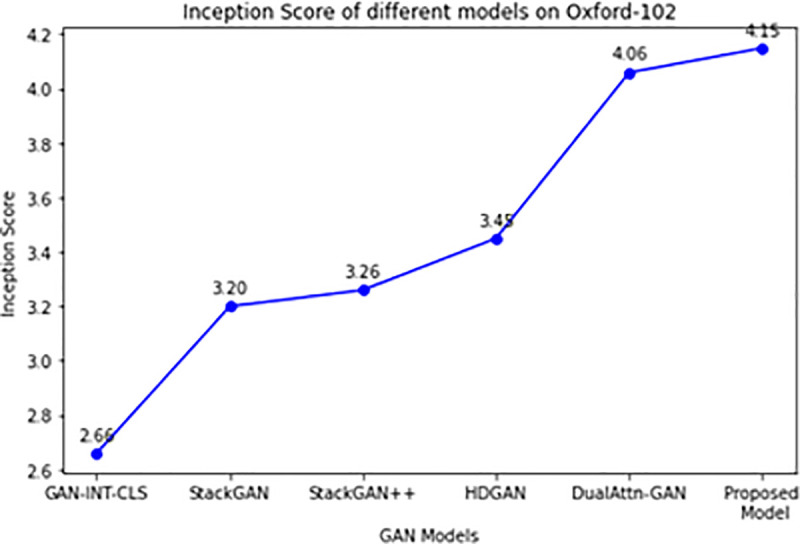
Inception Score on Oxford-102 flowers.

Fig 7 shows that IS of our model considerably surpasses GAN-INT-CLS by 1.49, StackGAN by 0.95, StackGAN++ by 0.89, HDGAN by 0.70, and DualAttn-GAN by 0.09 on Oxford-102 dataset. It indicates that our model can provide more distinct and easily understood images than earlier models. Furthermore, we also calculated the PSNR value, Fréchet Inception Distance (FID) and human rank for the proposed model. The FID quantitatively measures the similarity between the distribution of generated images and real images from a dataset, with lower scores indicating better performance. On the other hand, human rank captures the subjective judgment of human evaluators on the realism and fidelity of the generated images compared to the textual descriptions. Models achieving lower FID scores and higher human rank values are generally deemed more successful in producing high-quality images that closely align with the input text. Table 5 presents the evaluation of the proposed model on different metrics.

**Table 5:** Evaluation of the proposed method on different metrics.

Ref.	Model	Dataset	Method	PSNR	FID	HR
Proposed Model	RC-GAN	Oxford-102 Flowers	Deep Learning	**28.25 dB**	**12.34**	**Top 7%**
Proposed Model	RC-GAN	Oxford-102 Flowers (beta)	Deep Learning	**30.12 dB**	**10.56**	**Top 5%**

**Fig 8:**
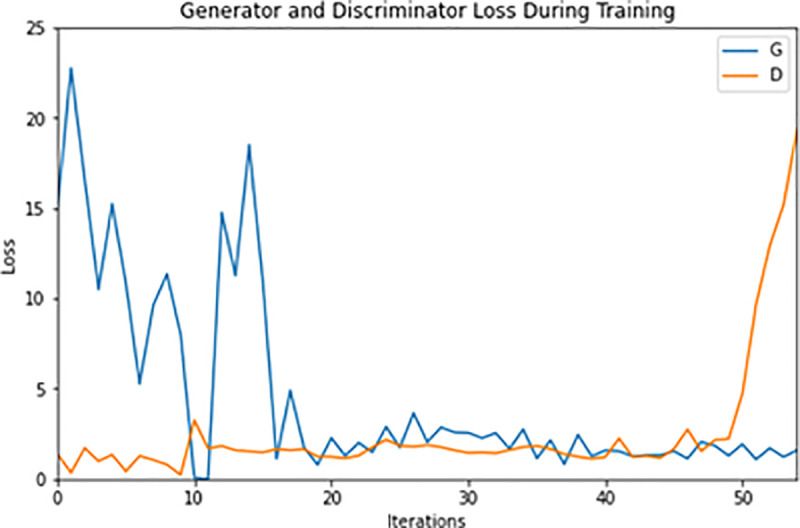
Generator and Discriminator Loss.

In GANs there are two loss functions, generator loss and discriminator loss. In start of the training, the loss of the generator is high, denoting that the generated image does not exactly matching with text input. At this time the loss of the discriminator model is low, its mean is that the discriminator is good for classifying that the image is not real it is fake. As the training proceeds the loss of the generator comes down with the quality of the images generated and the discriminator loss increase because it is not well at classifying the generated images. Fig 8 shows the generator loss and discriminator loss of our proposed model concerning the iterations during training.

## Conclusion

Text-to-image generation is a dominating research area nowadays within the domain of computer vision. It can be applicable for a variety of applications in multiple domains; crime investigation, photo-searching, art generation, portrait drawing, etc. In this research, we have used generative adversarial networks with RNN and CNN for generating images from text descriptions. We have used oxford-102 flowers dataset, which contains 8189 images divided into 102 classes. Originally, the dataset consisted of 10 text descriptions for every image, and our contribution is the modification of this dataset to a newer version oxford-102 flowers (beta). We expand the existing data and now it contains 15 text descriptions for each image. Moreover, we proposed a deep learning-based model for generating good-quality images according to given text input. We trained the model on both oxford-102 flowers and oxford-102 flowers (beta) datasets for generating images of 64 x 64, 128 x 128, and 256 resolutions. Inception score and PSNR are used for performance evaluation. Results from experiments show that the suggested GAN model performs more effectively than existing models by improving the best recorded IS by 4.15 on oxford-102 flowers of 64 x 64 dimensions, 3.87 on 256 x 256 resolutions and 3.97 on 128 x 128 oxford-102 flowers (beta) datasets. PSNR values are 28.25dB and 30.12dB on original and updated dataset.

## Supporting information

S1 File S1 Text: The petals on this flower are white and blue. S2 Text: The petals of this red flower are tightly coiled and gathered together. S3 Text: The flower with yellow center and white petals. S4 Text: This flower has pinkish color with yellow center and upward-folded petals. S5 Text: This flower features yellow petals with black inside lines. S6 Text: This flower has pink color long petals with an orange stamen cluster in the center. S7 Text: The petals on this flower have ruffled edges and are purple. S8 Text: This flower has a great many light green and white petals. S9 Text: Flowers having pink petals and darker center.
(ZIP)

## References

[1] Goodfellow IJ, Pouget-Abadie J, Mirza M, Xu B, Warde-Farley D, Ozair S, et al. Generative adversarial nets. Adv Neural Inf Process Syst. 2014;27.

[2] Reed S, Akata Z, Yan X, Logeswaran L, Schiele B, Lee H. Generative adversarial text to image synthesis. International conference on machine learning, 2016. 1060–9.

[3] Nilsback M-E, Zisserman A. Automated flower classification over a large number of classes. 2008 Sixth Indian Conference on Computer Vision, Graphics & Image Processing, 2008. 722–9. doi: 10.1109/icvgip.2008.47

[4] Wah C, Branson S, Welinder P, Perona P, Belongie S. The caltech-ucsd birds-200-2011 dataset.

[5] Lin TY, Maire M, Belongie S, Hays J, Perona P, Ramanan D, et al. Microsoft coco: Common objects in context. European conference on computer vision, 2014. 740–55.

[6] Khan MZ, Jabeen S, Khan MU, Saba T, Rehmat A, Rehman A. A realistic image generation of face from text description using the fully trained generative adversarial networks. IEEE Access. 2020;9:1250–60.

[7] Mishra P, Rathore TS, Shivani S, Tendulkar S. Text to image synthesis using residual GAN. 2020 3rd International conference on emerging technologies in computer engineering: Machine learning and internet of things (ICETCE), 2020. 139–44.

[8] Ouyang X, Zhang X, Ma D, Agam G. Generating image sequence from description with LSTM conditional GAN. 2018 24th International Conference on Pattern Recognition (ICPR), 2018. 2456–61. doi: 10.1109/icpr.2018.8545419

[9] Li B, Qi X, Lukasiewicz T, Torr PH. Manigan: Text-guided image manipulation. Proceedings of the IEEE/CVF Conference on Computer Vision and Pattern Recognition, 2020. 7880–9.

[10] Tao M, Tang H, Wu S, Sebe N, Jing XY, Wu F. Df-gan: deep fusion generative adversarial networks for text-to-image synthesis. arXiv. 2020. 6.

[11] Zhang C, Peng Y. Stacking VAE and GAN for context-aware text-to-image generation. 2018 IEEE Fourth International Conference on Multimedia Big Data (BigMM), 2018. 1–5.

[12] Zhang Z, Xie Y, Yang L. Photographic text-to-image synthesis with a hierarchically-nested adversarial network. Proceedings of the IEEE Conference on Computer Vision and Pattern Recognition, 2018. 6199–208.

[13] Li W, Zhang P, Zhang L, Huang Q, He X, Lyu S. Object-driven text-to-image synthesis via adversarial training. Proceedings of the IEEE/CVF conference on computer vision and pattern recognition, 2019. 12174–82.

[14] Girshick R. Fast R-CNN. Proceedings of the IEEE International Conference on Computer Vision, 2015. 1440–8.

[15] He K, Gkioxari G, Dollár P, Girshick R. Mask r-cnn. Proceedings of the IEEE international conference on computer vision 2017. 2961–9.

[16] Sharma S, Suhubdy D, Michalski V, Kahou SE, Bengio Y. Chatpainter: Improving text to image generation using dialogue. 2018. doi: 10.48550/arXiv.1802.08216

[17] Mao F, Ma B, Chang H, Shan S, Chen X. MS-GAN: Text to image synthesis with attention-modulated generators and similarity-aware discriminators. BMVC 2019. 150.

[18] Li W, Wen S, Shi K, Yang Y, Huang T. Neural architecture search with a lightweight transformer for text-to-image synthesis. IEEE Trans Netw Sci Eng. 2022;9(3):1567–76. doi: 10.1109/tnse.2022.3147787

[19] Yang Y, Wang L, Xie D, Deng C, Tao D. Multi-sentence auxiliary adversarial networks for fine-grained text-to-image synthesis. IEEE Trans Image Process. 2021;30:2798–809. doi: 10.1109/TIP.2021.3055062 33531300

[20] Omar M, Ur Rehman H, Samin OB, Alazab M, Politano G, Benso A. CapGAN: Text-to-Image Synthesis Using Capsule GANs. Information. 2023;14(10):552. doi: 10.3390/info14100552

[21] Tan YX, Lee CP, Neo M, Lim KM. Text-to-image synthesis with self-supervised learning. Pattern Recognition Letters. 2022;157:119–26. doi: 10.1016/j.patrec.2022.04.010

[22] Salimans T, Goodfellow I, Zaremba W, Cheung V, Radford A, Chen X. Improved techniques for training gans. Adv Neural Inf Process Syst. 2016;29.

[23] Zhang H, Xu T, Li H, Zhang S, Wang X, Huang X. Stackgan: Text to photo-realistic image synthesis with stacked generative adversarial networks. Proceedings of the IEEE International Conference on Computer Vision, 2017. 5907–15.

[24] Zhang H, Xu T, Li H, Zhang S, Wang X, Huang X, et al. StackGAN++: Realistic image synthesis with stacked generative adversarial networks. IEEE Trans Pattern Anal Mach Intell. 2019;41(8):1947–62. doi: 10.1109/TPAMI.2018.2856256 30010548

[25] Cai Y, Wang X, Yu Z, Li F, Xu P, Li Y. Dualattn-GAN: Text to image synthesis with dual attentional generative adversarial network. IEEE Access. 2019;7:183706–16.

[26] Cheng Q, Gu X. Hybrid attention driven text-to-image synthesis via generative adversarial networks. International Conference on Artificial Neural Networks. Cham: Springer International Publishing. 2019. 483–95

