# Supplementary figures and images for: Text-to-image generation with enhanced GANs: Bridging semantic gaps using RNN and CNN
Source: PLoS One. 2026 Jan 21;21(1):e0340413. doi: 10.1371/journal.pone.0340413 (PMC12822966; doi:10.1371/journal.pone.0340413)

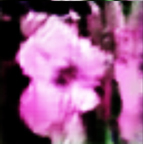

Supplement: S1 File — S1 Text: The petals on this flower are white and blue. S2 Text: The petals of this red flower are tightly coiled and gathered together. S3 Text: The flower with yellow center and white petals. S4 Text: This flower has pinkish color with yellow center and upward-folded petals. S5 Text: This flower features yellow petals with black inside lines. S6 Text: This flower has pink color long petals with an orange stamen cluster in the center. S7 Text: The petals on this flower have ruffled edges and are purple. S8 Text: This flower has a great many light green and white petals. S9 Text: Flowers having pink petals and darker center. (ZIP) [file pone.0340413.s001.zip › S9 Text 9.png]

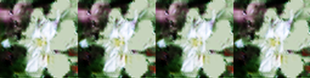

Supplement: S1 File — S1 Text: The petals on this flower are white and blue. S2 Text: The petals of this red flower are tightly coiled and gathered together. S3 Text: The flower with yellow center and white petals. S4 Text: This flower has pinkish color with yellow center and upward-folded petals. S5 Text: This flower features yellow petals with black inside lines. S6 Text: This flower has pink color long petals with an orange stamen cluster in the center. S7 Text: The petals on this flower have ruffled edges and are purple. S8 Text: This flower has a great many light green and white petals. S9 Text: Flowers having pink petals and darker center. (ZIP) [file pone.0340413.s001.zip › S8 Text 8.png]

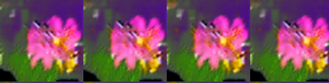

Supplement: S1 File — S1 Text: The petals on this flower are white and blue. S2 Text: The petals of this red flower are tightly coiled and gathered together. S3 Text: The flower with yellow center and white petals. S4 Text: This flower has pinkish color with yellow center and upward-folded petals. S5 Text: This flower features yellow petals with black inside lines. S6 Text: This flower has pink color long petals with an orange stamen cluster in the center. S7 Text: The petals on this flower have ruffled edges and are purple. S8 Text: This flower has a great many light green and white petals. S9 Text: Flowers having pink petals and darker center. (ZIP) [file pone.0340413.s001.zip › S7 Text 7.png]

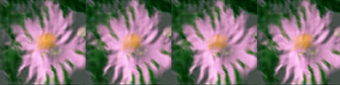

Supplement: S1 File — S1 Text: The petals on this flower are white and blue. S2 Text: The petals of this red flower are tightly coiled and gathered together. S3 Text: The flower with yellow center and white petals. S4 Text: This flower has pinkish color with yellow center and upward-folded petals. S5 Text: This flower features yellow petals with black inside lines. S6 Text: This flower has pink color long petals with an orange stamen cluster in the center. S7 Text: The petals on this flower have ruffled edges and are purple. S8 Text: This flower has a great many light green and white petals. S9 Text: Flowers having pink petals and darker center. (ZIP) [file pone.0340413.s001.zip › S6 Text 6.png]

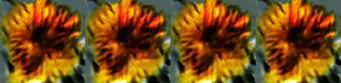

Supplement: S1 File — S1 Text: The petals on this flower are white and blue. S2 Text: The petals of this red flower are tightly coiled and gathered together. S3 Text: The flower with yellow center and white petals. S4 Text: This flower has pinkish color with yellow center and upward-folded petals. S5 Text: This flower features yellow petals with black inside lines. S6 Text: This flower has pink color long petals with an orange stamen cluster in the center. S7 Text: The petals on this flower have ruffled edges and are purple. S8 Text: This flower has a great many light green and white petals. S9 Text: Flowers having pink petals and darker center. (ZIP) [file pone.0340413.s001.zip › S5 Text 5.png]

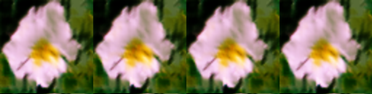

Supplement: S1 File — S1 Text: The petals on this flower are white and blue. S2 Text: The petals of this red flower are tightly coiled and gathered together. S3 Text: The flower with yellow center and white petals. S4 Text: This flower has pinkish color with yellow center and upward-folded petals. S5 Text: This flower features yellow petals with black inside lines. S6 Text: This flower has pink color long petals with an orange stamen cluster in the center. S7 Text: The petals on this flower have ruffled edges and are purple. S8 Text: This flower has a great many light green and white petals. S9 Text: Flowers having pink petals and darker center. (ZIP) [file pone.0340413.s001.zip › S4 Text 4.png]

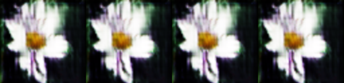

Supplement: S1 File — S1 Text: The petals on this flower are white and blue. S2 Text: The petals of this red flower are tightly coiled and gathered together. S3 Text: The flower with yellow center and white petals. S4 Text: This flower has pinkish color with yellow center and upward-folded petals. S5 Text: This flower features yellow petals with black inside lines. S6 Text: This flower has pink color long petals with an orange stamen cluster in the center. S7 Text: The petals on this flower have ruffled edges and are purple. S8 Text: This flower has a great many light green and white petals. S9 Text: Flowers having pink petals and darker center. (ZIP) [file pone.0340413.s001.zip › S3 Text 3.png]

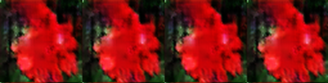

Supplement: S1 File — S1 Text: The petals on this flower are white and blue. S2 Text: The petals of this red flower are tightly coiled and gathered together. S3 Text: The flower with yellow center and white petals. S4 Text: This flower has pinkish color with yellow center and upward-folded petals. S5 Text: This flower features yellow petals with black inside lines. S6 Text: This flower has pink color long petals with an orange stamen cluster in the center. S7 Text: The petals on this flower have ruffled edges and are purple. S8 Text: This flower has a great many light green and white petals. S9 Text: Flowers having pink petals and darker center. (ZIP) [file pone.0340413.s001.zip › S2 Text 2.png]

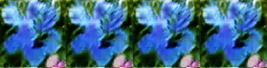

Supplement: S1 File — S1 Text: The petals on this flower are white and blue. S2 Text: The petals of this red flower are tightly coiled and gathered together. S3 Text: The flower with yellow center and white petals. S4 Text: This flower has pinkish color with yellow center and upward-folded petals. S5 Text: This flower features yellow petals with black inside lines. S6 Text: This flower has pink color long petals with an orange stamen cluster in the center. S7 Text: The petals on this flower have ruffled edges and are purple. S8 Text: This flower has a great many light green and white petals. S9 Text: Flowers having pink petals and darker center. (ZIP) [file pone.0340413.s001.zip › S1 Text 1.png]
